# Kinetic Properties and Pharmacological Modulation of High- and Low-Affinity Dopamine Transport in Striatal Astrocytes of Adult Rats

**DOI:** 10.3390/ijms25105135

**Published:** 2024-05-09

**Authors:** Vesna Sočan, Klemen Dolinar, Mojca Kržan

**Affiliations:** 1 Institute of Pharmacology and Experimental Toxicology, Faculty of Medicine, University of Ljubljana, 1000 Ljubljana, Slovenia; vesna.socan@mf.uni-lj.si; 2 Institute of Pathophysiology, Faculty of Medicine, University of Ljubljana, 1000 Ljubljana, Slovenia; klemen.dolinar@mf.uni-lj.si

**Keywords:** striatal astrocytes, adult rat, dopamine, dopamine uptake, DAT, NET, PMAT, OCT1

## Abstract

Astrocytes actively participate in neurotransmitter homeostasis by bidirectional communication with neuronal cells, a concept named the tripartite synapse, yet their role in dopamine (DA) homeostasis remains understudied. In the present study, we investigated the kinetic and molecular mechanisms of DA transport in cultured striatal astrocytes of adult rats. Kinetic uptake experiments were performed using radiolabeled [^3^H]-DA, whereas mRNA expression of the dopamine, norepinephrine, organic cation and plasma membrane monoamine transporters (DAT, NET, OCTs and PMAT) and DA receptors D1 and D2 was determined by qPCR. Additionally, astrocyte cultures were subjected to a 24 h treatment with the DA receptor agonist apomorphine, the DA receptor antagonist haloperidol and the DA precursor L-DOPA. [^3^H]-DA uptake exhibited temperature, concentration and sodium dependence, with potent inhibition by desipramine, nortriptyline and decynium-22, suggesting the involvement of multiple transporters. qPCR revealed prominent mRNA expression of the NET, the PMAT and OCT1, alongside lower levels of mRNA for OCT2, OCT3 and the DAT. Notably, apomorphine significantly altered NET, PMAT and D1 mRNA expression, while haloperidol and L-DOPA had a modest impact. Our findings demonstrate that striatal astrocytes aid in DA clearance by multiple transporters, which are influenced by dopaminergic drugs. Our study enhances the understanding of regional DA uptake, paving the way for targeted therapeutic interventions in dopaminergic disorders.

## 1. Introduction

Astrocytes, the most abundant glial cells in the central nervous system (CNS), are recognized for their diverse homeostatic functions, such as maintaining the blood–brain barrier, regulating ion balance, providing metabolic support to neurons and contributing to neuronal development and plasticity [1]. Protoplasmic astrocytes form peri-synaptic processes, establishing bidirectional communication with neurons in a structure named the tripartite synapse [2,3]. Astrocytes express various receptor and transporter proteins for neurotransmitters and possess the ability to release gliotransmitters themselves [4,5]. While their role in glutamate homeostasis is well established [6,7], their contribution to dopamine (DA) homeostasis remains an area of active investigation.

DA, essential for motor control, reward processing and cognition, exhibits regional specificity due to its distinct pathways, particularly in the human brain. The nigrostriatal pathway, originating in the substantia nigra pars compacta and projecting to the striatum, plays a critical role in motor control [8]. Disruptions in this pathway, primarily DA depletion in the striatum due to the degeneration of dopaminergic neurons in the substantia nigra, are implicated in Parkinson’s disease (PD) [9,10]. Astrocyte activation, a process defined as reactive astrogliosis, has been linked to the progression of PD [10,11]. While the role of astrocytes in PD has been explored [12], their specific contributions to DA clearance, synthesis, storage and responses to DA and dopaminergic drugs in the striatum remain enigmatic.

DA exerts its physiological effects by binding to metabotropic G-protein-coupled DA receptors, which are classified into two subtypes: D1 and D2 receptors [8,13]. The concentration of DA within the synaptic cleft is primarily regulated through its reuptake mechanism, predominantly facilitated by the sodium-dependent, low-capacity, high-affinity dopamine transporter (DAT) [8]. Additionally, other transporters contribute to the regulation of DA levels, albeit to a lesser extent. Notably, studies on rodent models have indicated that the norepinephrine transporter (NET) exhibits comparable affinity for DA and assumes a more prominent role in DA uptake within brain regions where DAT expression is limited, notably in the cortex [14]. Moreover, sodium-independent transporters characterized by a low affinity for DA but a high capacity for transport (referred to as uptake 2), such as organic cation transporter 3 (OCT3) [15,16] and the plasma membrane monoamine transporter (PMAT) [17,18], play a significant role in removing excess extracellular DA, particularly when its concentration exceeds the capacity of the DAT [19].

Unlike electrically excitable neurons, astrocytes respond to DA with transient intracellular calcium fluctuations [20,21,22], but the underlying mechanisms have not been explored extensively. The presence of all DA receptor subtypes on astrocytes has been reported, albeit with inconsistencies across species and brain regions [23,24,25]. Dopaminergic drugs like clozapine, haloperidol [26] and apomorphine [27] influence DA receptor mRNA expression in astrocytes, particularly apomorphine, which has been observed to induce the release of trophic factors from astrocytes [28]; however, the underlying mechanisms of this process need further exploration.

Astrocytes contribute to DA uptake in various brain regions. Striatal astrocytes, cultured from neonatal Sprague-Dawley rats [29] and neonatal BALB/c mice [30], appear to express the primary DA transporter (DAT). Interestingly, cortical DA uptake has been observed to involve the NET rather than the DAT [27,31,32]. While uptake 1 transporters such as the DAT and NET are crucial drug targets, and therefore are an intriguing area of research, astrocyte DA clearance likely heavily relies on the polyspecific transporters (i.e., uptake 2). Uptake 2 transporters involved in DA uptake, such as the PMAT [18] and the OCTs [16], characterized by their high capacity but low affinity for DA, utilize the inside-negative membrane potential as a driving force and are independent of the Na^+^ and Cl^-^ ion gradient [18]. Uptake 2 serves as a sort of a backup system in monoamine clearance and is of particular importance when DA exceeds the capacity of the high-affinity transporters [19].

Whether astrocytes solely act as a backup system for DA clearance or represent a potential therapeutic target in DA-related disorders remains to be elucidated. Uncovering the intricate mechanisms of astrocytic DA handling and modulation, while considering regional variations, holds immense potential for understanding and therapeutically targeting DA-related neuropathologies.

## 2. Results

### 2.1. Kinetic Experiments of [^3^H]-DA Uptake in Striatal Astrocytes of Adult Rats

#### 2.1.1. Temperature and Time Dependence of [^3^H]-DA Uptake

**Figure 1:**
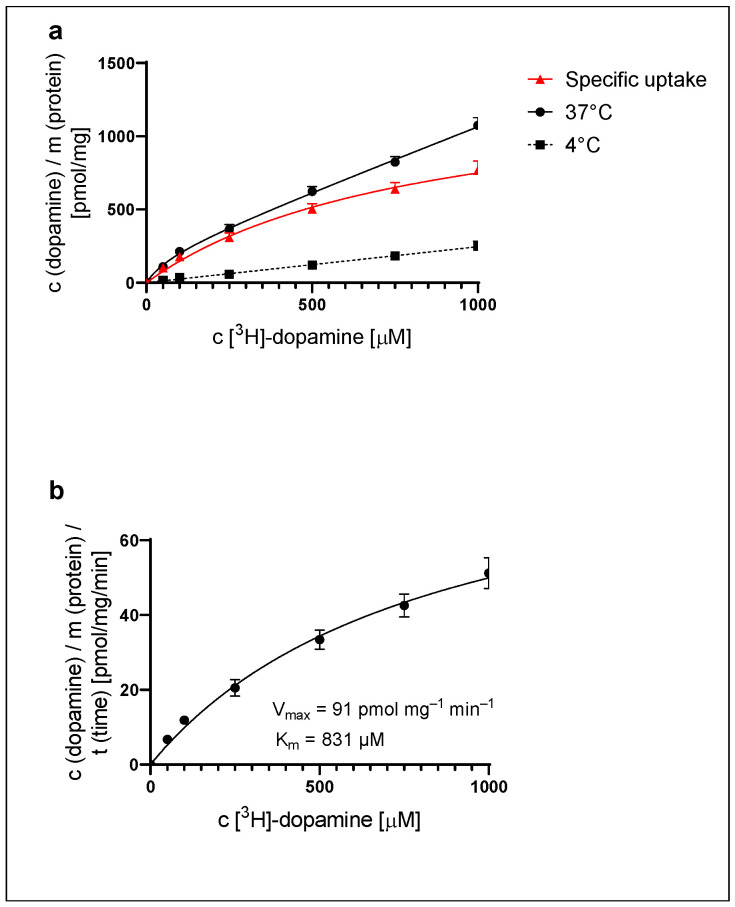
[^3^H]-DA uptake in striatal astrocytes of adult rats: (**a**) total (37 °C), non-specific (4 °C) and specific uptake of [^3^H]-DA and (**b**) uptake velocity of [^3^H]-DA, presented as mean ± SEM of two separate experiments (*n* = 6). Total and non-specific uptake were significantly different at each tested concentration (unpaired *t*-test, *p* < 0.05).

To investigate the ability of astrocytes to take up DA, striatal astrocytes of adult rats were exposed to a 0.03–1000 µM concentration of [^3^H]-DA for a time span of 15 min, the latter determined by prior experiments [27]. [^3^H]-DA uptake was temperature- and concentration-dependent (Figure 1a). Total (37 °C) and non-specific (4 °C) [^3^H]-DA uptake was significantly different at each tested concentration (*p* < 0.05). Specific [^3^H]-DA uptake, calculated as the difference between total and non-specific uptake, appeared saturable with the apparent B_max_ calculated at 1372 ± 192 pmol/mg. DA uptake velocity (Figure 1b) was calculated from the specific uptake and the time span of [^3^H]-DA incubation (15 min). The kinetic parameters of uptake velocity were calculated using the Michaelis–Menten equation. The apparent maximal uptake rate of DA, V_max_, was calculated as 91 ± 13 pmol/mg/min, and the apparent Michaelis–Menten constant, K_m_, was calculated as 831 ± 209 µM.

#### 2.1.2. Sodium Dependency and Inhibition by Transporter Inhibitors D22, GBR12909, Desipramine and Nortriptyline of [^3^H]-DA Uptake

**Figure 2:**
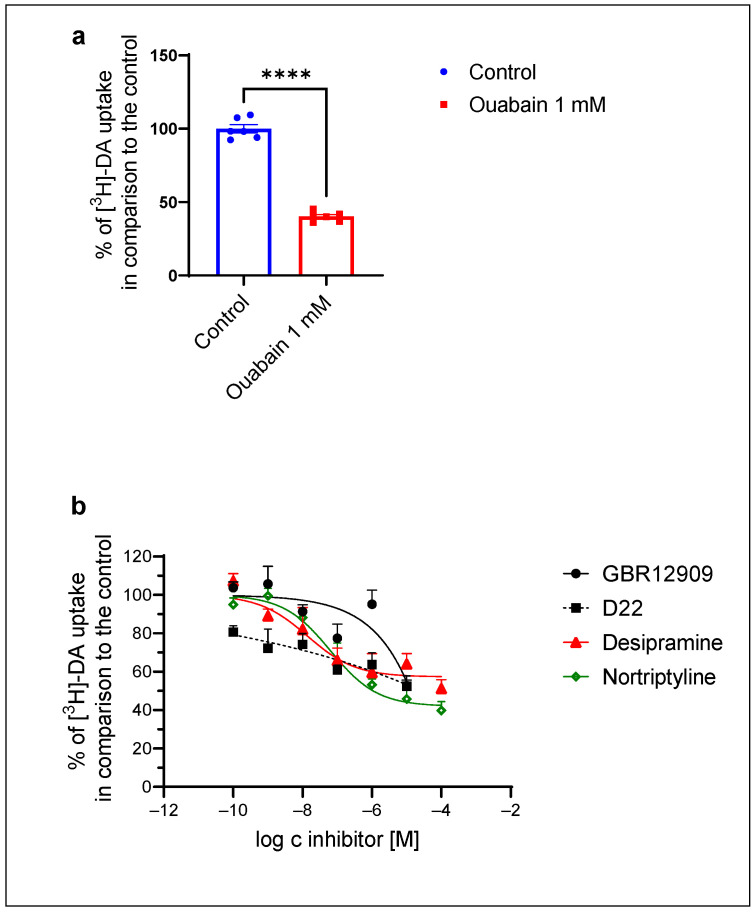
Inhibition of [^3^H]-DA uptake in striatal astrocytes of adult rats by (**a**) Na^+^/K^+^-ATPase inhibitor ouabain. Results are presented as percentage of control (mean ± SEM) of two separate experiments (*n* = 6); unpaired *t*-test: **** *p* < 0.0001. (**b**) Inhibition of [^3^H]-DA uptake by antidepressants desipramine and nortriptyline, DAT inhibitor GBR12909 and D22 in striatal astrocytes of adult rats presented as percent of control (mean ± SEM) from three separate experiments (*n* = 9).

To investigate whether striatal astrocytes possess active DA carrier systems, dependent on the gradient of sodium ions, we measured DA uptake in the presence of the Na^+^/K^+^-ATPase inhibitor ouabain (Figure 2a). The 30 nM [^3^H]-DA uptake was significantly reduced to 40 ± 1% in the presence of the Na^+^/K^+^-ATPase inhibitor (1 mM) (Figure 2a), indicating the involvement of active transporters dependent on the gradient of Na^+^ concentration in striatal astrocyte DA uptake.

Additionally, we investigated the effects of various monoamine uptake inhibitors on [^3^H]-DA uptake (Figure 2b). Striatal astrocyte DA uptake was significantly reduced by the NET selective inhibitors desipramine (IC_50_ = 0.014 ± 0.014 µM, pIC_50_ = 7.8) and nortriptyline (IC_50_ = 0.064 ± 0.040 µM, pIC_50_ = 7.2). D22, an inhibitor of the high-capacity, low-affinity DA transporters such as the PMAT and the OCTs, significantly reduced (unpaired *t*-test, *p* < 0.05) DA uptake in comparison to the control to 81 ± 3% at a 10^−10^ M and to 52 ± 5% at a 10^−5^ M concentration; however, the IC_50_ could not be calculated from the inhibition curve. GBR12909, a DAT-selective inhibitor, did not induce significant inhibition of the 30 nM [^3^H]-DA uptake (Figure 2b).

### 2.2. mRNA Expression of Transporters Involved in DA Uptake in Striatal Astrocytes of Adult Rats

**Figure 3:**
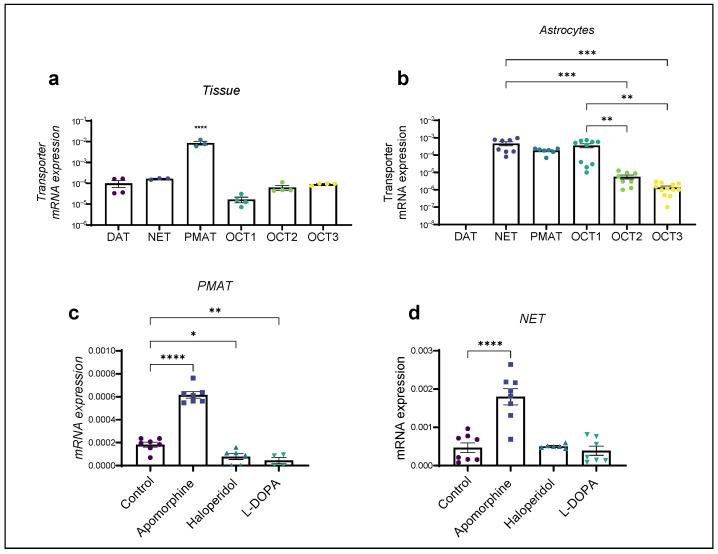
mRNA expression of (**a**) transporters DAT, NET, PMAT, OCT1, OCT2 and OCT3 in striatal tissue of adult rats (One-Way ANOVA with post hoc Tukey’s correction, **** *p* < 0.0001); (**b**) transporters DAT, NET, PMAT, OCT1, OCT2 and OCT3 in striatal astrocytes of adult rats (One-Way ANOVA with post hoc Dunnett’s test (F(4,40) = 9.5, *p* < 0.0001), *** *p* < 0.001, ** *p* < 0.05); and changes in mRNA expression of (**c**) PMAT and (**d**) NET after 24 h exposure to apomorphine, haloperidol and L-DOPA. Data are presented relative to expression of endogenous control, β-actin, as mean ± SEM of at least two separate experiments. Statistical analysis of changes in mRNA expression after exposure to dopaminergic compounds apomorphine, haloperidol and L-DOPA was performed using One-Way ANOVA with post hoc Dunnett’s test (NET F (3,25) = 22.06, *p* < 0.0001, PMAT F(3,20) = 106.9, *p* < 0.0001); (**c**) **** *p* < 0.0001, ** *p* = 0.0073, * *p* < 0.05; (**d**) **** *p* < 0.0001.

Kinetic experiments in the present study indicate the involvement of both uptake 1 and uptake 2 DA carrier systems in striatal astrocytes of adult rats. To further explore which transporters may be involved in astrocyte DA uptake, we performed a qPCR analysis of the mRNA of transporters DAT, NET, PMAT, OCT1, OCT2 and OCT3 in both tissue samples and astrocyte cultures from the striatum of adult rats. Striatal tissue exhibited statistically significantly greater expression of PMAT (One-Way ANOVA with Tukey’s correction for multiple comparisons (F(5,16) = 31.36, *p* < 0.0001), whereas the level of expression of the DAT, the NET and the OCTs OCT1, OCT2 and OCT3 was lower. Among the OCTs, OCT3 expression was the most prominent. Striatal tissue exhibited similar levels of expression of the active DA uptake transporters DAT and NET (Figure 3a). Striatal astrocytes of adult rats exhibited a distinct transporter mRNA expression profile (Figure 3b). DAT expression could not be detected by our qPCR method. Contrary to the striatal adult rat tissue NET, PMAT and OCT1 expression was similar. The NET and OCT1 mRNA expression was significantly greater than OCT2 and OCT3.

Astrocyte cultures were exposed to a 100 µM concentration of apomorphine, haloperidol and L-DOPA for 24 h (Figure 3c,d). Viability of astrocyte cultures after exposure to these drugs was measured previously [27,32]. Astrocyte mRNA expression of the NET was upregulated only by apomorphine, whereas PMAT mRNA expression was affected by all three dopaminergic compounds. Apomorphine induced a significant upregulation of mRNA expression of both the studied transporter receptors and conversely induced a downregulation of the PMAT.

### 2.3. mRNA Expression of DA Receptors D1 and D2 in Striatal Astrocytes of Adult Rats

**Figure 4:**
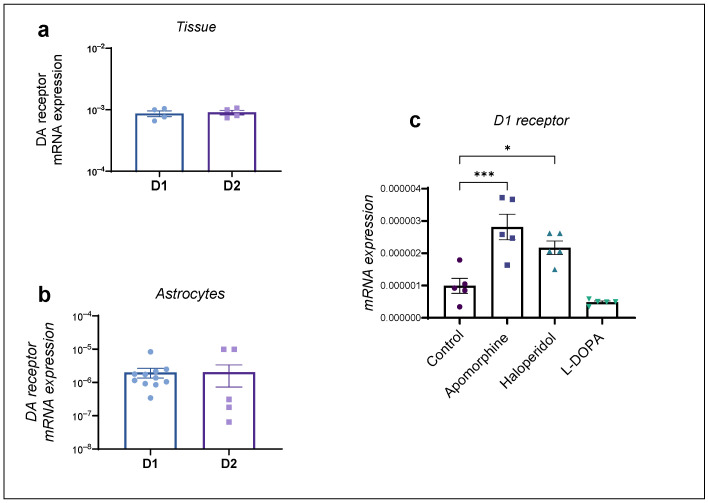
DA D1 and D2 mRNA expression in adult rat striatal (**a**) tissue (*n* = 4) (unpaired *t*-test, *p* > 0.05), (**b**) astrocytes (*n* = 11) (unpaired *t*-test, *p* > 0.05) and (**c**) changes in mRNA expression of DA receptor D1 after 24 h exposure to dopaminergic drugs apomorphine, haloperidol and L-DOPA. Data are presented relative to expression of endogenous control, β-actin, as mean ± SEM of at least two separate experiments. Statistical analysis of changes in mRNA expression after exposure to dopaminergic compounds apomorphine, haloperidol and L-DOPA was performed using One-Way ANOVA with post hoc Dunnett’s test (F(3,16) = 17.78, *p* < 0.0001), *** *p* = 0.0003, * *p* < 0.05.

We additionally investigated mRNA expression of the DA receptors D1 and D2 in the striatal tissue and astrocytes of adult rats and changes in DA receptor mRNA expression after a 24 h exposure to dopaminergic drugs. The striatal tissue exhibited prominent expression of both subtypes of DA receptors (Figure 4a), whereas the striatal astrocytes of adult rats exhibited low, albeit similar, levels of both DA receptors, D1 and D2 mRNA expression (Figure 4b). The expression of the D2 receptor was at the limit of detection of our qPCR method and could not be detected in multiple samples (which were included in the statistical analysis as 0); therefore, further analysis included only D1 receptor mRNA expression.

Astrocyte cultures were exposed to the dopaminergic compounds apomorphine, haloperidol and L-DOPA, as described above. We observed a significant upregulation of the DA receptor D1 in the presence of apomorphine and haloperidol, whereas L-DOPA induced a modest downregulation of the DA D1 receptor, which was, however, not statistically significant (Figure 4c).

## 3. Discussion

In the present study, we investigated the mechanisms of DA uptake in striatal astrocytes of adult Wistar rats. Our findings suggest that unlike neurons, which primarily utilize the DAT [33] for DA uptake, astrocytes employ a complex system involving multiple transporters, which may not involve the DAT. In previous work, our research group investigated the characteristics of DA transport in the striatal and cortical astrocytes of neonatal rats, as well as the cortical astrocytes of adult rats of the same strain [27,32]. Studies show that astrocytes are a heterogenous rather than homogenous group of cells [34] that are not only species but also brain-region specific. Therefore, our aim was to elucidate the brain region specificity of astrocyte DA uptake. Additionally, we were interested whether astrocytes respond to treatment with dopaminergic drugs, such as apomorphine, haloperidol and L-DOPA, to investigate whether they may serve as a possible therapeutic target or if we could elucidate the possibility of fine-tuning existing dopaminergic treatments.

Astrocytes have been shown to contribute to the clearance of DA from the synapse by multiple studies [31,35,36,37,38,39,40,41,42]; however, the intricacies of the regional and species-specific characteristics of DA uptake appear to be unclear. Most previous studies have observed DA uptake in cortical astrocytes of neonatal rats [31,35,36,40] and a few in astrocyte cultures from neonatal rat striatum [37,38,43]. To the best of our knowledge, no studies have observed DA in the striatal astrocytes of adult rats. Some studies have reported that astrocytes contribute to the DA clearance largely by diffusion [37,38], without the involvement of active transporters; others have observed temperature-dependent DA uptake as well sodium dependency [39,40] and sensitivity to transporter inhibitors such as the potent NET inhibitor nisoxetine [31,35], the tricyclic antidepressant desipramine [31], the OCT and PMAT inhibitor D22 [31], the serotonin transporter inhibitor zimelidine [35], and the DAT inhibitors GBR-12909 [35] and GBR-12935 [31] in cortical astrocytes of neonatal rats.

In the present study, our findings indicate a temperature and concentration dependence of striatal astrocyte DA uptake, suggesting the involvement of a transporter-mediated carrier system. Although saturable, the capacity of astrocytes to take up DA is at the limit of the observed highest transient concentrations of DA in the synapse, which are presumed to be in the low millimolar range [44]. The potent inhibition of a 30 nM DA uptake by ouabain, a Na^+^/K^+^-ATPase inhibitor, indicates the presence of an active carrier system in the striatal astrocytes of adult rats. Multiple studies have reported similar findings [39,40]; however due to low capacity of the high-affinity monoamine transporters, at concentrations of DA in the micromolar range, low-affinity, high-capacity transporters such as the PMAT and the OCTs become of crucial importance [45]. These findings suggest, similarly to our research on astrocytes obtained from the neonatal cortex and striatum, as well as cortical astrocytes of adult rats, the involvement of both uptake 1 and uptake 2 transporters.

The significance of the DAT in effective DA clearance and its role as a pharmacological target is well established. While some studies report DAT expression in striatal astrocytes [29,30], results vary across brain regions and species. The results of Inazu et al. [31] on cortical astrocytes of neonatal rats as well as results from our research group on cortical astrocytes of neonatal and adult Wistar rats [27,32] indicate that astrocyte DA transport may be mediated by the NET rather than the DAT. Our findings suggest prominent NET mRNA expression in the striatal astrocytes of adult rats compared to the DAT. Notably, the NET inhibitors desipramine and nortriptyline showed potent inhibition of DA uptake compared to the DAT inhibitor GBR12909. These findings indicate that adult rat striatal and cortical DA uptake may be mediated by similar carrier systems involving the NET rather than the DAT.

Uptake 2 transporters are crucial for monoamine uptake, especially when the capacity of the uptake 1 transporters is exceeded or their function is impeded. Of the OCTs, OCT2 and OCT3 are more prominently expressed in the CNS [19,46], and their presence has been observed in astrocytes [44,46]. OCT1 expression in the CNS is low, and given its relatively low expression in the brain compared to the other uptake 2 transporters, it seems unlikely to be a key driver of monoamine homeostasis [44]. However, the qPCR analysis of striatal astrocyte cultures in the present study revealed prominent mRNA expression of OCT1, with OCT3 scarcely present. The PMAT, only discovered in 2004 [17,18], was prominently expressed both in striatal tissue as well as striatal astrocytes. The overlapping substrate selectivity between the PMAT and OCTs makes it difficult to distinguish the relative contribution of each transporter to monoamine uptake [19,47]. D22 has been observed to inhibit the OCTs; however, it is more selective towards the PMAT [48] (pKi 7.0 [18,49]). The potent inhibition of DA uptake by D22 suggests that striatal astrocyte DA transport may rely heavily on the PMAT; however, it may additionally to some extent be mediated by OCT1 or OCT2.

Several studies have demonstrated the ability of astrocytes to take up the DA precursor L-DOPA [29,50,51,52,53,54], alongside expressing DA receptors in their membrane [55,56,57,58]. Astrocytes respond to DA with intracellular calcium fluctuations [59,60] and may participate in responses to dopaminergic drug treatment, such as DA receptor antagonists (e.g., haloperidol and clozapine) and agonists (e.g., apomorphine) [28,61,62,63,64,65,66]. In the present study, we observed comparable mRNA expression levels of the DA receptors D1 and D2, albeit at low levels. Despite DA receptors being the primary pharmacological targets of dopaminergic drugs like apomorphine and haloperidol, dopaminergic drugs may induce a wide array of effects in astrocytes [28,61,63,64,65,67,68,69,70]. Apomorphine, for instance, has been observed to enhance the biosynthesis of various trophic growth factors and promote neuronal survival [28], whereas haloperidol and L-DOPA have been associated with inducing proinflammatory responses, particularly in the case of L-DOPA-induced dyskinesia linked with glial activation [65,71,72].

In the present study, we observed that PMAT mRNA expression was most significantly altered by all three dopaminergic drugs, similarly to our research on cortical astrocyte cultures of adult rats, whereas in neonatal rat astrocytes cultivated from striatum, of the three compounds, only apomorphine induced the upregulation of the NET, and not the PMAT. Haloperidol has been found to change the expression of neuronal D1 and D2 receptors [73,74,75]; however, studies report inconsistent results. In the present study, we observed a downregulation in mRNA expression of the PMAT and an upregulation of the DA receptor D1 in the presence of haloperidol. Although it is a DA receptor D2 antagonist, haloperidol has been found to inhibit hPMAT activity at micromolar concentrations [76]. These findings indicate that astrocytes exhibit changes in mRNA expression when treated with dopaminergic drugs, particularly apomorphine and haloperidol. For a more comprehensive understanding, further studies are needed to investigate varying drug concentrations and incubation times. Additionally, it is crucial to assess potential protein-level changes, such as up- or downregulation, to better understand the impact of these drugs on astrocyte function.

It is important to acknowledge the limitations of our study. While our findings provide valuable insights into the mechanisms of DA uptake in the striatal astrocytes of adult rats, more research is needed to clarify the molecular processes driving transporter-mediated astrocyte DA clearance. Additionally, the application of these findings to in vivo models and clinical settings has yet to be explored.

Our study did not focus on serotonin transporter (SERT)-mediated DA uptake, which we recognize as a potential limitation. Given the intricate relationship between DA and norepinephrine systems, particularly in areas such as the striatum, further examination of the SERT’s role in DA transport could deepen our understanding of astrocyte function. Future research using knockout approaches could help distinguish the contributions of each transporter more precisely, offering a robust and detailed understanding of their respective roles.

In conclusion, our study contributes to a deeper understanding of the complex mechanisms governing DA uptake in the striatal astrocytes of adult rats. By unraveling the distinct transporter profile of striatal astrocytes and highlighting the role of transporters such as the NET and PMAT, our findings pave the way for future research aimed at developing targeted therapeutic interventions for dopaminergic disorders.

## 4. Materials and Methods

### 4.1. Materials

All cell culture reagents except fetal bovine serum (FBS, Cambrex IEP GmbH (Wiesbaden, Germany)) were obtained from Gibco, Invitrogen (Paisley, UK). [^3^H]-dopamine (2220 GBq/mmol) was purchased from Perkin Elmer (Hopkinton, MA, USA), an E.Z.N.A.^®^ Total RNA Kit I from Omega Bio-Tek (Norcross, GA, USA), and a High Capacity cDNA Reverse Transcription Kit, TaqMan Gene Expression Assays and TaqMan^®^ Universal PCR Master Mix from Applied Biosystems (Carlsbad, CA, USA). Decynium-22 (D22), nortriptyline HCl, apomorphine HCl, haloperidol, L-DOPA, L-deprenyl HCl and tropolone were obtained from Sigma Aldrich (St. Louis, MO, USA), and desipramine HCl was obtained from Sandoz (Cham, Switzerland). GBR12909 was obtained from Tocris (Bristol, UK).

### 4.2. Animals and Primary Cell Culture Preparation

For the purpose of our study, we obtained the permission of the Administration of the Republic of Slovenia for Food Safety, Veterinary and Plant Protection issue U34401-20/2017/2. This study was approved by the National Veterinary Administration (approval numbers U34401-23/2022/6, approval date 20 June 2017, and U34401-23/2022/6, approval date 23 December 2022). We used brains from redundant (ten in total) sexually mature rats, Rattus norvegicus, breed Wistar, of both genders, weighing 180–200 g, and all procedures complied with the relevant Slovenian and European legislation. Animals, sacrificed for the purpose of removing organs (brains), were euthanized by decapitation after anesthesia with CO_2_, which was assessed as the most suitable method. All necessary measures were used for the prevention of unnecessary suffering and discomfort of the laboratory animals used. Striatal tissue of adult rats was excised whole and either flash-frozen or used for cell culture preparation. Cultured rat striatal astrocytes were prepared from adult rat striata by a well-established protocol, routinely used in our laboratory [77]. Astrocyte cultures were grown and maintained in T-75 flasks (Falcon, Corning, NY, USA) and 12-well plates (Falcon). Briefly, cells were grown in high-glucose Dulbecco’s modified Eagle’s medium (DMEM) containing 10% FBS, 1 mM pyruvate, 2 mM glutamine and 25 µg/mL gentamycin/streptomycin in a humidified 95% air–5% CO_2_ atmosphere. Confluent cultures were shaken at 225 RPM overnight, the medium was changed the next morning, and this process was repeated a total of three times. After the third overnight shaking, the cells were trypsinized, washed in DMEM and subcultured into 12-well plates; the medium was changed each week from then on. After three weeks, the cultures contained 93–100% type 1 astrocytes (up to 7% consisting of the remaining microglia, neuronal cells, fibroblasts and endothelial cells) [78].

### 4.3. [^3^H]-Dopamine Uptake Experiments

#### 4.3.1. Dependence of [^3^H]-DA Uptake on Temperature and Concentration

Monolayer cultures in 12-well plates were washed twice in the uptake buffer containing CaCl_2_ (25 mM HEPES, 125 mM NaCl, 4.8 mM KCl, 1.2 mM KH_2_PO_4_, 1.2 mM MgSO_4_, 1.4 mM CaCl_2_ and 5.6 mM glucose, pH 7.4) and monoamine oxidase (tropolone, 0.1 mM) and catechol-O-methyltransferase (L-deprenyl, 0.1 mM) inhibitors at 37 °C (total uptake) and at 4 °C (non-specific uptake). The uptake buffer was added to each well in addition to [^3^H]-DA, with a concentration ranging from 0.03 to 1000 µM, resulting in a total volume of 500 µL. The uptake time of the experiments was determined previously as 15 min [27]. After 15 min the buffer was removed, and the plates were washed four times with ice-cold uptake buffer without Ca^2+^. The cells were lysed in 300 µL of 0.5 M NaOH. Then, 250 µL of each sample was transferred to a scintillation vial, and the radioactivity was measured. From 4 µL of each sample, we measured the protein concentration using the Bradford method using the Bio-Rad Protein Assay (Hercules, CA, USA).

#### 4.3.2. Inhibition of [^3^H]-DA Uptake by Desipramine, Nortriptyline, GBR12909, D22 and Ouabain

For the inhibition studies, monolayer cultures in 12-well plates were prepared in the same manner as described previously and preincubated in different test compounds at various concentrations (10^−8^–10^−3^ M) for 20 min then incubated with an [^3^H]-DA concentration of 30 nM for 15 min at 37 °C. The chosen inhibitors of transporters were desipramine, nortriptyline, GBR12909 and D22. The reaction was stopped by placing the plates on ice and washing with ice-cold uptake buffer without Ca^2+^ in the same manner as described above. The cells were lysed by the addition of 0.5 M NaOH, samples were collected and DA uptake was determined in the same manner as described above. Ouabain (Na^+^/K^+^-ATPase inhibitor) sensitivity was tested by a 20 min preincubation of cell plates, followed by a 15 min incubation with 30 nM [^3^H]-DA. The same steps were then followed as described above.

### 4.4. mRNA Expression of Transporters and Receptors by Quantitative Polymerase Chain Reaction (qPCR)

Total RNA was extracted from adult rat striatal astrocyte cell cultures as well as striatal tissue samples using the E.Z.N.A.^®^ Total RNA Kit I. An amount of 1 μg of RNA was used to synthesize cDNA utilizing a High-Capacity cDNA Reverse Transcription Kit, according to the manufacturer’s instructions. qPCR was performed using TaqMan™ Universal PCR Master Mix and TaqMan Assays, according to the manufacturer’s instructions, in a QuantStudio^TM^ 3 System (Applied Biosystems, MA, USA): SLC6A3 (Rn00562224_m1), SLC6A2 (Rn00580207_m1), SLC22A1 (Rn00562250_m1), SLC22A2 (Rn00580893_m1), SLC22A3 (Rn00570264_m1), SLC29A4 (Rn01453824_m1), DA receptor D1 (Rn03062203_s1), DA receptor D2 (Rn00561126_m1) and ß-actin (Rn00667869_m1). The expression of target genes was normalized to the expression of ß-actin according to the equation [target/reference] = [EFFreference^Cqreference^]/[EFFtarget^Cqtarget^], where Cq is the quantification cycle and EFF is the amplification efficiency (expressed as a value between 1 and 2). EFF was determined with LinRegPCR software (Version 2014.7) [79].

### 4.5. Cell Culture Treatment

Confluent three-week-old adult rat striatal astrocyte cell cultures plated in 12–well plates were treated with three different compounds, haloperidol, apomorphine and L-DOPA, for 24 h. The concentration of drugs used for the cell treatment was set at 100 µM, based on cell viability experiments of prior studies [27,32].

### 4.6. Data Analysis

The uptake experiments were routinely carried out in triplicates or quadruplicates, and each experiment was repeated at least twice. All data are reported as arithmetic means ± SEM. From the cell cultures in the 12-well plates, 3 wells were used as a triplicate or 4 wells as a quadruplicate in each experiment, which was then repeated at least twice or three times using different cell cultures cultivated from different animals. Data and statistical analysis were performed in consideration to each experiment with GraphPad Prism 9.5. software (San Diego, CA, USA). Th kinetic parameter V_max_ was determined by the Michaelis–Menten equation, and IC_50_ and pIC_50_ were calculated from the log(inhibitor) vs. response—Variable slope (four parameters) equation using GraphPad Prism software. Data normality was assessed by the Shapiro–Wilk test. A comparison between the means of two groups was carried out using the unpaired *t*-test. A comparison between multiple groups of data was performed using an Ordinary One-Way ANOVA with post hoc Dunnett’s or Tukey’s correction. Differences were considered significant at *p* < 0.05.

## Data Availability

All data are contained within the manuscript. The data presented in this study are available on request from the corresponding author.
